# Influenza Virus Infection during Pregnancy as a Trigger of Acute and Chronic Complications

**DOI:** 10.3390/v14122729

**Published:** 2022-12-07

**Authors:** Osezua Oseghale, Ross Vlahos, John J. O’Leary, Robert D. Brooks, Doug A. Brooks, Stella Liong, Stavros Selemidis

**Affiliations:** 1 School of Health and Biomedical Sciences, RMIT University, Bundoora, VIC 3083, Australia; 2 Discipline of Histopathology, School of Medicine, Trinity Translational Medicine Institute (TTMI), Trinity College Dublin, DO8 XW7X Dublin, Ireland; 3 Sir Patrick Dun’s Laboratory, Central Pathology Laboratory, St James’s Hospital, D08 XW7X Dublin, Ireland; 4 CERVIVA Research Consortium, Trinity College Dublin, DO8 XW7X Dublin, Ireland; 5 Clinical and Health Sciences, University of South Australia, Adelaide, SA 5001, Australia

**Keywords:** pregnancy, influenza A virus, vascular dysfunction, preeclampsia

## Abstract

Influenza A virus (IAV) infection during pregnancy disrupts maternal and fetal health through biological mechanisms, which are to date poorly characterised. During pregnancy, the viral clearance mechanisms from the lung are sub-optimal and involve hyperactive innate and adaptive immune responses that generate wide-spread inflammation. Pregnancy-related adaptations of the immune and the cardiovascular systems appear to result in delayed recovery post-viral infection, which in turn promotes a prolonged inflammatory phenotype, increasing disease severity, and causing maternal and fetal health problems. This has immediate and long-term consequences for the mother and fetus, with complications including acute cardiopulmonary distress syndrome in the mother that lead to perinatal complications such as intrauterine growth restriction (IUGR), and birth defects; cleft lip, cleft palate, neural tube defects and congenital heart defects. In addition, an increased risk of long-term neurological disorders including schizophrenia in the offspring is reported. In this review we discuss the pathophysiology of IAV infection during pregnancy and its striking similarity to other well-established complications of pregnancy such as preeclampsia. We discuss general features of vascular disease with a focus on vascular inflammation and define the “Vascular Storm” that is triggered by influenza infection during pregnancy, as a pivotal disease mechanism for short and long term cardiovascular complications.

## 1. IAV Infection during Pregnancy and Maternal and Fetal Vulnerability

IAV infection of humans during seasonal epidemics and pandemics results in significant mortality and morbidity [1]. Seasonal influenza epidemic patterns often occur biannually with large and intense outbreaks that lead to considerable health and economic burdens [2]. The World Health Organisation (WHO) estimates that at least 5–10% of the world’s population is infected in seasonal epidemics each year, resulting in 290,000 to 650,000 deaths [3]. This morbidity and mortality impact of seasonal influenza often involves a secondary bacterial infection such as pneumonia [4,5]. Viral infection with concomitant pneumonia directly leads to about a 10% fatality rate in the 3–5 million patients with influenza A reported each year [1]. Furthermore, the economic burden of IAV infections is enormous, with for example the US alone spending an estimated $10.4 billion a year on flu-related expenses [6]. Influenza pandemics occur intermittently and are usually due to major alterations in the viral composition of a seasonal strain of IAV [1]. Cumulatively, four major IAV pandemics have occurred in the past century; the Spanish flu (H1N1) pandemic, occurring between 1918–1919, the H2N2 Asian influenza (1957–1958) and the 1968 Hong-Kong influenza (H3N2), cumulatively resulting in 108 million fatalities world-wide [1,7]. Furthermore, the 2009 H1N1 swine flu pandemic accounted for 280,000 deaths [8]. Influenza can be deadly to specific groups in the human population, owing to the elevated risk of complications developing from the initial infection. The Centres for Disease Control and Prevention (CDC) list children less than 5 years of age, the elderly, healthcare workers and pregnant women as the highest-risk populations. Additionally, individuals with underlying medical conditions including cardiovascular diseases (CVD) and cancer are included in the list of high risk individuals [6].

In pregnancy, the vulnerability of the mother and fetus to IAV infection is significantly elevated [9] and both seasonal and pandemic influenza can have a significant impact on the health of the mother and fetus [10]. Notably, seasonal IAV infection is associated with a spike in peri-natal mortality with miscarriages, as well as early neonatal disease and death [11,12]. Indeed, the emergence of cardiopulmonary comorbid adaptive changes in pregnancy, alterations in heart rate, stroke volume and reduced pulmonary residue capacity, are understood to contribute to the severity of IAV-driven morbidity and mortality in the mother [12,13,14]. Pandemic IAV outbreaks in the last century had detrimental effects on the pregnant population [15] with interactions between virus and the host immune system having an impact on immunogenicity and pathogenesis [16]. The pathophysiological mechanisms underlying the increased risk of IAV infection in pregnant women and the fetus are not fully understood [17]. Increased exposure to seasonal epidemics and reluctance to treat pregnant women with category C drugs, due to adverse effects observed in fetal animal studies, has been suggested as a primary reason for increased complications and disease [18]. Category C drugs are drugs that either have no controlled studies in animals to reveal their adverse effects on the fetus (teratogenic) or lack controlled studies in women to show its safety. However, their potential benefits may warrant the use of the category C drugs in pregnancy despite the risk they pose [19]. Some examples include, gentamicin, amlodipine, fluoroquinolones, and saccharin [19]. To address the significant problem of IAVs impact on maternal and fetal health, there is an urgent need to better understand the pathophysiology of viral infection during pregnancy. 

### 1.1. The Innate Immune Response to IAV Infection in the Lung

**Figure 1:**
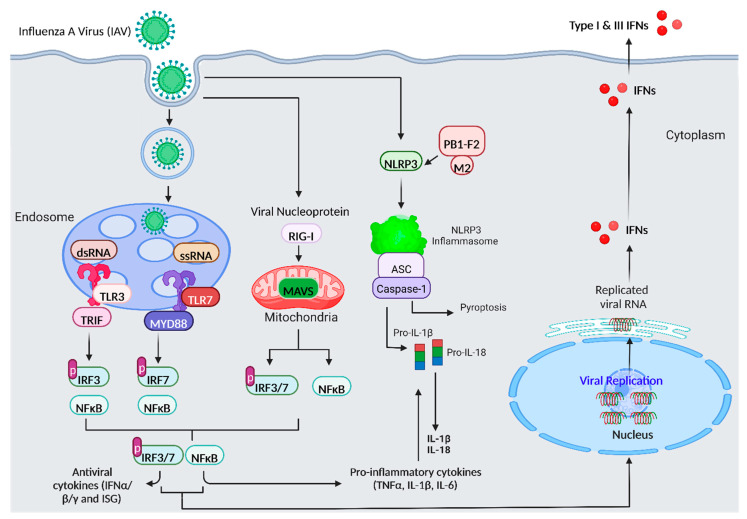
**Schematic diagram for the innate immune response against influenza A virus (IAV) infection.** The intracellular detection of IAV occurs by host pathogen recognition receptors (PRRs) such as TLR3 and TLR7. TLR3 and TLR7 signalling then activates transcription factor: nuclear factor kappa-light-chain-enhancer of activated B cells (NF-κB) via adaptor proteins TIR-domain-containing adapter-inducing interferon-β (TRIF) and myeloid differentiation factor 88 (MyD88), respectively. Transcription factors: IRF3 and IRF7 are also activated for TLR3 and TLR7, respectively. NF-κB functions to activate the transcription of pro-inflammatory cytokines such as tumour necrosis factor (TNF-α), interleukin 6 (IL-6) and interleukin (IL)-1β, while IRFs trigger the activation of type I interferons (IFN α and β) and IFN-stimulated genes (ISG). The recognition of viral RNA in the cytosol by retinoic acid-inducible gene-I protein (RIG-I) leads to the induction of IFN-β and ISG through mitochondria antiviral signalling (MAVS) protein and IRF3. Moreover, IAV activates NOD-like receptor pyrin domain containing 3 (NLRP3) is activated by PB1-F2 and M2 ion channel leading to an increased expression of NLRP3 inflammasome. Pro-IL-1 and pro-IL-18 are cleaved into mature IL-1 and IL-18 by the inflammasome, a multi-protein complex that first attracts pro-caspase-1 through ASC (the adaptor molecule apoptosis-associated speck-like protein). Inflammasome activation also promotes pyroptosis, an inflammatory form of cell death. ”p” denotes phosphorylation.

The innate immune system functions by mounting an inherent defence against infectious agents, which then initiates adaptive immunity and in turn establishes immunological memory [20]. Innate immune activation often begins via a process known as pathogen sensing, which involves pattern recognition receptors (PRRs) including retinoic acid-inducible gene-I protein (RIG-I) like receptors, toll-like receptors (TLR) and NOD-like receptors that recognise pathogen-associated molecular patterns (PAMPs) expressed by an infectious agent [21,22]. Recognition of pathogens by plasmacytoid dendritic cells (pDCs) generates a cascade of TLR signalling and this involves sensing of for example, double stranded RNA (dsRNA) by TLR3 and single stranded RNA (ssRNA) by TLR7 [23]. Downstream signalling of TLR7 occurs via the adaptor protein myeloid differentiation factor 88 (MyD88), which functions in the endocytosis and activation of nuclear factor kappa-light-chain-enhancer of activated B cells (NF-κB) and type 1 interferonregulatory factor 7 (IRF7) [23,24]. NF-κB stimulates the expression of pro-inflammatory cytokines such as interleukin (IL)-1β, IL-6 and tumour necrosis factor (TNF-α), while IRF7 increases the expression of anti-viral cytokines including type I IFNs and interferon stimulated genes (ISG) to limit viral replication [25,26]. In addition, TLR3 in airway epithelial cells, DCs and macrophages, induces signalling via adaptor protein TIR-domain-containing adapter-inducing interferon-β (TRIF), which activates transcription factors NF-κB and IRF3 to concomitantly induce pro-inflammatory cytokines and type I IFNs expression [20]. The NOD-like receptor pyrin domain containing 3 (NLRP3) protein complex, which consists of NLRP3 inflammasomes and NLR apoptosis inhibitory proteins, including pro-caspase-1 and apoptosis-associated speck-like protein (ASC), are also activated after IAV infection [27,28]. NLRP3 inflammasomes can be activated by IAV, which involves an M2 ion channel and PB1-F2 response, together with increased activation of NF-κB, the secretion of IL-1β and IL-18, plus increased oxidative stress, reduced autophagy and this leads to lung injury [29,30]. However, blocking the NLRP3 inflammasome has been observed to limit viral replication and inflammation. Indeed, by combining oseltamivir and sirolimus (mTOR inhibitor) viral replication can be blocked and the mTOR-NLRP3-IL-1β axis suppressed, which can however mediate immune lung damage [28] (Figure 1). IAV primarily infects the alveoli and epithelial layer of the respiratory tract, leading to the activation of tissue-resident alveolar macrophages, which can then phagocytose IAV infected cells [16]. The endocytosis of IAV and sensing of viral RNA by for example TLR7 results in the activation of nicotinamide adenine dinucleotide phosphate-oxidase 2 (NOX2) (Vlahos et al., 2011; Vlahos et al., 2013; To et al., 2017) and production of pro-inflammatory cytokines such as TNF-α, which can both contribute adversely to IAV-induced lung pathology [31]. In addition, antigen-presenting cells (APC) such as resident lung dendritic cells (DCs) within the airway epithelium barrier function to facilitate viral clearance by monitoring the airway lumen and presenting the infected cell virions to helper and cytotoxic T cells for destruction [32]. Consequently, while the innate immune system is essential for combatting IAV infection, the virus has adapted to this defence mechanism and can modulate the immune response to generate an uncontrolled inflammatory response that favours viral replication and can cause lung pathogenesis.

Local lung infection by IAV leads to monocyte and leukocyte activation that generates reactive oxygen species (ROS) including hydrogen peroxide (H_2_O_2_), hydroxyl (OH^.^) radical and peroxynitrite (ONOO^−^), and prooxidant cytokines such as TNF-α [33,34]. Oxidative stress triggered by the pro-inflammatory milieu in the lungs involves an imbalance in oxidant and antioxidant levels that IAV manipulates in favour of an oxidant environment in infected cells and surrounding tissue. This oxidant phenotype ensues from the failure of antioxidant mechanisms to neutralise oxidants, thus resulting in exacerbated pathologies [35]. In influenza mouse models there is a marked increase in superoxide (O_2_^−^) producing cells and H_2_O_2_ formation within the bronchoalveolar lavage fluid (BALF) in the lung, indicative of oxidative stress. Furthermore, evidence attributes the source of pathogenic ROS to the NOX2 oxidase enzyme, which ensues from TLR7 recognition of viral RNA. Indeed, TLR7 dependent NOX2 oxidase activation drives ROS in endosomes. This is a key feature of IAV infection, and uncontrolled endosomal ROS production contributes directly to IAV pathology [36,37]. The dysregulation of ROS production by IAV infection during pregnancy correlates directly with host morbidity and involves decreased cellular responses, together with altered cytokine and Th1 cell responses [38,39,40]. IAVs capacity to evoke a Th1 cellular response is critical for generating a pro-inflammatory environment and also involves a reduction in the anti-inflammatory Th2 cellular response [41]. In addition, the percentage of viral-limiting pDCs are reduced in pregnancy when compared to non-pregnant individuals [16], which further skews the immune response in favour of IAV. The levels of key cytokines including interleukin (IL)-6, IL-12 and TNF-α are consequently elevated due to the impaired recruitment of type 1 interferons (IFN) and peripheral blood mononucleated cells (PBMCs) by respiratory epithelial cells [42,43]. Viruses like IAV have therefore evolved to manipulate the innate immune response to cause exacerbated inflammation and to limit the recruitment of the adaptive immune system, but this effect and the susceptibility of the host is much more pronounced during pregnancy.

### 1.2. The Adaptive Immune Response to IAV in Lung

**Figure 2:**
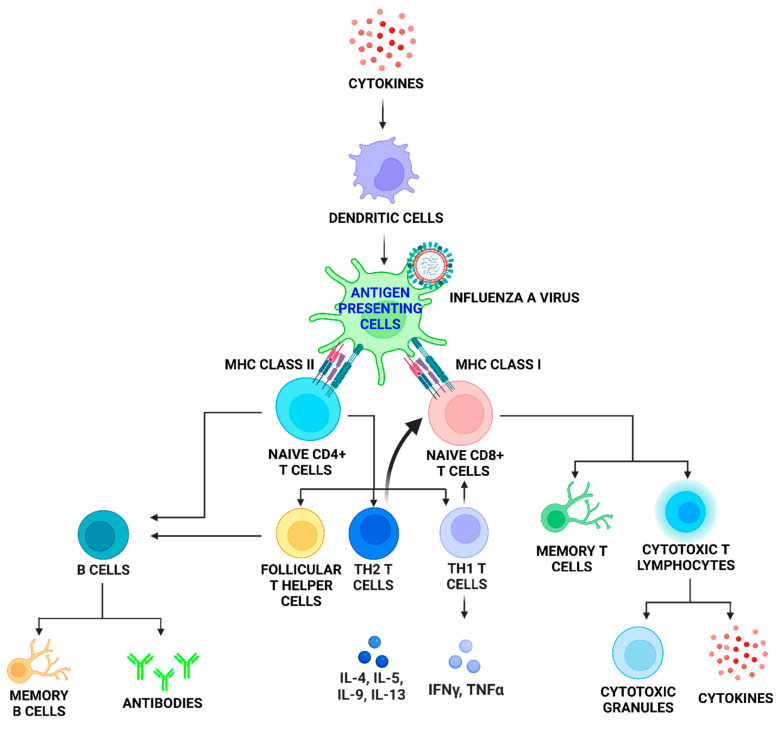
**Schematic diagram of the adaptive immune response to influenza A virus infection (IAV).** Following the detection of IAV, the innate immune response utilises dendritic cells (DCs) to produce interferons (IFNs) and cytokines, which facilitate DC maturation into antigen presenting cells (APCs) as part of the adaptive immune response recruitment process, to initiate immune T and B cell responses. The activation of antigen bearing DCs triggers the differentiation of naïve CD4^+^ T cells into its subsets; Th1, Th2, and follicular helper T cells (TfH). Follicular helper T cells activate B cells which later form long-lived memory B cells and assist in generating antibodies. Th1 and Th2 T cells are capable of regulating CD8^+^ T cell differentiation and indirectly help in the maintenance of its response. CD8^+^ T cells undergo rapid expansion, differentiation and migration to infected cells following activation by DCs to form immunological memory T cells. In addition, within the infected cells, cytotoxic T lymphocytes response is initiated prior to secreting cytokines and cytotoxic granules that induce apoptosis.

The adaptive immune response to influenza infection involves the recruitment of CD4^+^ or CD8^+^ T cells and immunoglobulin producing B cells to limit replication and effect viral clearance [44]. CD4^+^ T cell lymphocytes are activated by DCs migrating from the lungs to draining lymph nodes during IAV infection, and are a key component of the antiviral immune response [16,45]. CD4^+^ T cells generally differentiate into Th1 cells in response to IAV infection and express antiviral cytokines such as TNF-α, IFN-γ and activate alveolar macrophages [46,47]. CD4^+^ T cells can function independently of PAMP recognition, to directly regulate innate inflammation within the first 48 h of IAV infection as well as CD8^+^ T cell responses by secreting pro-inflammatory cytokines [48,49]. CD8^+^ T cells utilise a variety of effector mechanisms to eradicate virally infected cells and limit viral replication. Most notable is the production of pro-inflammatory mediators (i.e., TNF, IFN-γ and macrophage inflammatory protein 1-alpha (MIP-1α)), the direct destruction of infected cells by perforin/granzyme and pro-apoptotic TNF receptor family dependent mechanisms [49,50,51]. Upon IAV infection, naïve CD8^+^ T cells become activated by DCs that migrate from the lung to the draining lymph nodes and in the process lead to T cell proliferation and differentiation into cytotoxic T lymphocytes (CTLs) [31]. The differentiation and proliferation into CTLs also involves IFN-γ and pro-inflammatory cytokines that are generally increased by CD4^+^ T cells [49,52]. CTLs are suggested to upregulate the expression of C-X-C motif chemokine receptor 3 (CXCR3) and C-C motif receptor 4 (CCR4) while concomitantly decreasing the expression of CCR7. This occurs, so as to facilitate immune cell migration from the lymph nodes back into the lungs to kill IAV infected cells [20]. CD8^+^ T cells are also suggested to have a protective function during influenza infection, as the dysregulation of CD8^+^ T cells contribute to the development of influenza-induced immunopathology [53,54]. This protective role stems from the formation of memory CTL cells post IAV clearance, that are fast in their response to secondary IAV infection [55] (Figure 2). These IAV-specific CD8^+^ T cells are demonstrated to survive for up to 2 years in murine models [56]. Studies on local lung adaptive immunity in response to influenza infection during pregnancy have been limited, possibly due to the preconceived notion that the local lung adaptive immune response is similar in pregnant and non-pregnant cohorts. However, the very dramatic changes in innate immunity during IAV infection must have a significant effect on the recruitment of adaptive immunity and is therefore an area of immunity that is in need of further analysis.

### 1.3. Systemic Inflammatory Response to IAV Infection

Systemic inflammation during IAV infection encompasses modifications that occur across the entire maternal and fetal landscape, including for example the dissemination of pro-inflammatory signals via antiviral conditioning of bone marrow-derived leukocytes, and the activation of antigen-presenting memory T cells [57]. The systemic inflammation that occurs in pregnant women in many respects is different when compared to non-pregnant individuals due to the presence of fetal antigens [58]. IAV-driven systemic immune manifestations are dependent on hematopoietic stem cells (HSCs) derived from the bone marrow and these produce leukocytes and monocytes [57]. During an infection, bone marrow-derived HSCs are primed by IFNs to mount a local and systemic antiviral immune response [59]. For example, following intravenous injection of IFN-α in mice with a systemic infection, dormant HSCs become activated and the levels of leukocytes increases, which in turn leads to a dampening of systemic inflammation [60]. 

In pregnancy, to limit the prevalence of autoimmune attack on the fetus, several specific immune adaptations are initiated, but these adaptations underpin the increase in disease severity to IAV infection, the delay in recovery and the reduction in CD4^+^ and CD8^+^ T cells in the blood [16]. In lipopolysaccharide (LPS) inoculated pregnant CD-1 mice, the systemic inflammation involves several dynamic and functional differences in maternal systemic immunity when compared to non-pregnant animals. For example, serum concentrations of IL-10, IL-12, IL-6 and TNF-α are increased, with associated decreases in immune reactivity and an increase in maternal predisposition to infection [61]. These same changes to immune cells and cytokines occur in the maternal systemic immunity during IAV infection and in addition, prolonged systemic inflammation is common in influenza-infected pregnant women. This correlation has been made in several studies, for example, in pregnant BALB/c mice infected with pandemic H1N1 09 virus, serum cytokines levels was observed to increase as early as 2 days post IAV infection [62]. Although serum cytokine levels increased at day 2 post infection, little is known about the timepoint when systemic inflammation begins to subside in pregnancy. There are significant health implications that arise from the altered innate and adaptive immune response to IAV in the lung of the mother that extend systemically in both the mother and fetus.

## 2. Immediate and Long-Term Health Complications Associated with IAV Infection in Pregnancy

Seasonal and pandemic IAV infection during pregnancy alters pregnancy-driven adaptations via systemic inflammation that ensues following local respiratory insult. Several immediate and long-term health complications accompany the local lung and systemic inflammation following IAV infection. This milieu in turn triggers significant alterations in the fetus without IAV directly infecting fetal tissues nor the placenta. 

### 2.1. Maternal Complications

Seasonal IAV has led to significant spikes in hospitalisation rates and increased mortality amongst pregnant women [14,63], and this involves respiratory failure and acute cardiopulmonary disease [64,65]. In hospitalised pregnant patients with IAV infection, there is a five-fold increase in influenza-induced complications. [66]. In the US alone, during the 1918 Spanish flu pandemic, of the ~1350 confirmed cases of influenza infection in pregnant women 27% died from the infection. Following the development of secondary bacterial pneumonia, the death rate in IAV infected pregnancy patients increased significantly [67,68]. Autopsy reports suggested that the onset of pulmonary oedema created an environment conducive for secondary bacterial pneumonia development, which potentially explains the subsequent increases in mortality [67,68].

#### 2.1.1. Secondary Bacterial and Viral Pneumonia

Two of the most common complications occurring from influenza infection are the development of viral (commonly varicella-zoster virus) and bacterial (*Streptococcus pneumoniae*) pneumonia. Patients initially present with acute respiratory distress, then a decrease in pulmonary functional residual capacity and cell-mediated immunity [69]. The rate of pneumonia is similar between pregnant and non-pregnant individuals; however, the morbidity and mortality are greater for pregnant patients. Thus, it is imperative to offer higher surveillance and intervention for pregnant women as there is an increased risk of fetal complications [70]. 

The 1957 and 1968 influenza pandemics resulted in a 20% mortality rate for pregnant women, prompting the classification of pregnancy as a risk factor for severe disease from IAV infection [71]. The most recent IAV pandemic, the 2009 A(H1N1)pdm09 further highlighted the notion that pregnancy increased the risk for the development of severe disease. The data suggests that intensive care unit (ICU) admissions, influenza-induced complications, and death, are higher in pregnant women when compared to non-pregnant individuals [72]. Moreover, pre-existing conditions such as asthma, diabetes, and hypertension, are likely to exacerbate the risk of death from influenza infection. Pregnant women with asthma and other respiratory complications represented 43.5% of the recorded mortality from pandemic IAV infection, and this equates to the exacerbated viral pathogenesis that occurs in asthmatic adults [73].

#### 2.1.2. Acute Respiratory Distress Syndrome

ARDS is a causative factor for the respiratory malfunction associated with multiple organ failure, and is characterised by increased pulmonary oedema, impaired carbon dioxide excretion and hypoxemia [74]. Moreover, inflammation, increased platelet, and leukocyte activity, altered alveolar endothelial and epithelial barrier permeability and an uncontrolled activation of coagulation pathways are characteristic immune responses associated with ARDS. Although rare in normal pregnancy, in complicated pregnancies such as PE or during IAV infections, the ARDS mortality rates in pregnancy range from 11% to 50% [75]. It is important to note that there is little distinction between ARDS in pregnancy and non-pregnant individuals in term of disease severity. However, it appears that complications of the physiological adaptations that occur in pregnancy are likely to predispose pregnant women to increased morbidity or mortality.

Although studies implicate the local inflammatory response during viral infection to the level of morbidity and mortality in pregnancy, a key knowledge gap is the systemic inflammatory mechanisms involved. Thus far, our comprehension of IAV infection in pregnancy suggests that systemic inflammation can proffer markedly debilitating effects to the fetus as well as the mother, hence the need to further examine the impact of influenza-induced systemic inflammation during pregnancy.

### 2.2. Fetal Complications from Maternal IAV Infection

Adverse fetal complications during maternal IAV infection do not appear to be the result of vertical or transplacental transmission of IAV as these events are rare. However, highly pathogenic strains of IAV such as the avian influenza A (H5N1) may still transfer to the placenta [9]. Low pathogenic IAV strains may negatively impact placenta health via the stimulation of apoptosis, and viral replication. For example, a study by Quang Duy Trinh, et al. observed that challenging first trimester trophoblast cell lines including Swan71 and HTR8 cells with seasonal IAV strain, H3N2 A/Udorn/72 resulted in significant intracellular localisation of the viral proteins and increased apoptosis via a direct cytopathic effect of IAV [76]. A notable observation was the unchanged expression of human leukocyte antigen (HLA)-G and a rejection from maternal immune system [76]. Furthermore, despite the placenta being spared from low pathogenic seasonal IAV strain (HKx31) infection and replication, IAV triggered an increase in hypoxia gene expression in the placenta. The hypoxia is likely the result of an impairment in the maternal cardiovascular system [77,78]. Highly pathogenic pandemic IAV strains appear to be more susceptibile to the immunological barrier mounted by trophoblast cells within the placenta. Indeed, Komine-Aizawa S, et al. demonstrated that first trimester trophoblast cell lines Swan71 and HTR8 challenged with H1N1 A/Narita/2009 strain inhibited the release of the virus into trophoblast cells despite their susceptibility to the virus [79]. The attenuation of viral release and cytopathic effects by the immunological barrier of the trophoblast cell lines meant that the damage to placenta was minimal [79]. The combination of pneumonia, and acute respiratory syndrome may contribute to the downstream fetal developmental issues [80]; with a systemic immune response that may be driven by both viral RNA and for example, bacterial derived LPS. Research into the impact of maternal IAV infection on fetal health is limited, though some notable observations have been made. These observations include, but are not limited to increases in preterm birth, neonatal deaths, miscarriages and fetal growth retardation/restriction (FGR/IUGR) [81,82,83]. The impact of maternal IAV infection on the development of FGR and neurodevelopmental disorders such as schizophrenia will be discussed. The limitations in the research into the link between maternal IAV infection, rare vertical transmission, and fetal neurodevelopmental disorders will be addressed. In addition the potential link between vascular impairment, limited placental perfusion and the inception of fetal neurological developmental disorder will be highlighted.

#### 2.2.1. Fetal Growth Retardation or Intrauterine Growth Restriction

FGR is secondary to placenta dysfunction and is associated with a range of adverse outcomes, whereas IUGR refers to an impairment in growth and development of the embryo/fetus and/or its organs during pregnancy [84]. Changes in the placental vasculature are important during FGR and IUGR, due to the critical functional exchange between the mother and fetus, involving the supply and transport of respiratory gases, nutrients, and waste removal [85]. IUGR occurs in response to reduced utero-placental blood flow and angiogenesis, and this results in inefficient blood supply to the fetus. Seasonal influenza epidemics may increase the risk of adverse fetal outcomes, but there are conflicting publications, and the association is not well defined. Data from a 13-year population-based cohort study in Nova Scotia suggests that influenza in pregnancy leads to offspring that are small for gestational age (SGA) and respiratory illness in infants when compared to the control group [86]. During the 2009 influenza pandemic in Norway, there was an increase in the risk of fetal death in pregnant women infected with the pandemic strain (hazard ratio, 1.91; 95% confidence interval, 1.07 to 3.41) [87]. In contrast, data from the Tennessee Medicaid program showed no differences in SGA offspring and respiratory illness parameters for infants born to pregnant women hospitalised for pre-existing respiratory illnesses such as asthma, compared with matched controls during the influenza season [88]. While there is inconclusive evidence for the impact of growth restriction and IAV on pathogenic outcome, this apparent disparity could possibly be explained by a common mechanism(s) of detrimental effect in the fetus.

#### 2.2.2. Schizophrenia (Neurodevelopmental Disorder)

Neurological disorders in offspring from complicated pregnancies, is an issue of significant health care concern. Evidence suggest that the altered vascular function in hypertensive pregnancies can initiate neurological disorders in the setting of viral infection [89,90,91]. The direct vascular inflammatory insult and elevation in blood pressure (BP) that occurs during hypertension, is efficient in impairing vascular relaxation and may potentially limit blood and nutrient supply to the fetus [91]. Indeed, this limitation is known to occur in pregnancy induced hypertension (PIH), such as PE and gestational hypertension, which are both known to be associated with a higher risk of schizophrenia in the offspring in later life [92,93]. Several processes have been proposed to influence the extent and severity of fetal neurodevelopmental disorders from maternal infection. Genetic susceptibility, duration of infection and placental inflammation caused by a spike in pro-inflammatory markers in maternal circulation are some of the likely factors contributing to fetal neurodevelopmental disorders [94,95].The development of schizophrenia is observed mainly in adolescence and adult offspring due to the mechanistic pathophysiological abnormalities in placental blood perfusion [92]. Moreover, higher levels of anxiety, depression and withdrawal are reported in offspring born to women with gestational hypertension [96]. In animal studies, offspring from IAV/NWS/33 H1N1 infected pregnant mice, displayed behavioural alterations similar to the pathology of schizophrenia and/or autism [97,98]. Brain abnormalities were reported in neonate and adult offspring including cerebellum atrophy and reduced fractional anisotropy in the corpus collosum at postnatal day 14 in the neonate, and right middle cerebellar peduncle fractional anisotropy at postnatal day 56 in the adult offspring [99]. Despite the evidence from animal studies, recent evidence from systematic reviews have found no association between exposure to maternal IAV infection at any trimester during pregnancy and the development of schizophrenia or developmental delays [100]. This reiterates the need for more research into the correlation between IAV infection in pregnancy and downstream neuropsychiatric outcomes.

## 3. Critical Adaptations in Pregnancy

In normotensive pregnancy, immunological and cardiovascular adaptations occur that support both maternal health and fetal development. Immunological adaptation is thought to involve immunosuppression to limit fetal rejection [14], while cardiovascular adaptations are required to support adequate nutrients and blood supply for fetal development [101,102]. These biological adaptations are targeted by IAVs impact on immune system regulation with the collateral effect of enhancing disease progression and severity in the mother and fetus. Progesterone is critically important in maintaining a pregnancy, but the altered hormone environment coincides with physiological changes that suit and can be exploited by IAV. For example, progesterone is a muscle relaxant and leads to airway dilation that may aid viral dissemination and access to lower regions of the lung where inflammation can result in secondary bacterial infection and pneumonia. In addition, progesterone interacts with PGE2 to increase vascular permeability in the lungs, and this may give IAV vascular access and enable further dissemination, which in turn provokes systemic immunity in the mother. Finally, progesterone can influence the innate immune system to reduce NK-cell regulation and increase neutrophil chemo-attractants in the lung which could exacerbate inflammation.

### 3.1. Immunological Adaptations in the Mother and Fetus

The placenta and fetus trigger complex changes in the maternal immune system, and this immunomodulation is important in maintaining a viable pregnancy [58]. These immune alterations can occur specifically at the uterine mucosal level and also in the peripheral immune system [103]. Immune tolerance needs to be established and maintained throughout pregnancy to tolerate the presence of fetal antigens in the mother’s circulatory system [103]. During pregnancy, cell-mediated (T-helper type I) and humoral (T-helper type II) immunity are altered to promote fetal antigen tolerance. 

Cell-mediated immunity is evoked by pro-inflammatory cytokines including IFN-α, IL-2, and TNF-a,β. Moreover, lymphocyte recognition of cell-associated foreign antigens is critical in the defence against invading intracellular pathogens including influenza viruses [58]. Humoral immunity is controlled by anti-inflammatory cytokines including IL-4 and IL-10 and are utilised to promote maternal fetal antigen tolerance [104]. In pregnancy, the Th1/Th2 balance is skewed towards a Th2-associated immunity to promote an immunosuppressed state. This bias is largely owed to hormonal factors, and macrophages at the maternal-fetal interface that predominately release Th2-stimulating cytokines [105]. The Th2 bias leads to a suppression in CTLs, a stimulation of B-lymphocytes [106], and an augmentation in antibody-mediated immunity [105]. Clinically, this alteration may be observed in pregnant patients with signs of autoimmune disease and pre-eclampsia [107]. With up to 70% of women with rheumatoid arthritis experiencing temporal remission during pregnancy while disease flares are observed in women with systemic lupus erythematosus due to excessive autoantibody production [108,109]. During IAV infection in pregnancy, the opposite effect is required, with a more pronounced Th1 phenotype necessary for viral elimination [16]. The dynamic balance between immune suppression and an effective immune response to pathogen infection is exploited by IAV to create a proinflammatory environment that limits effective immunity while promoting viral replication and pathogenesis.

### 3.2. Cardiovascular Adaptations

**Figure 3:**
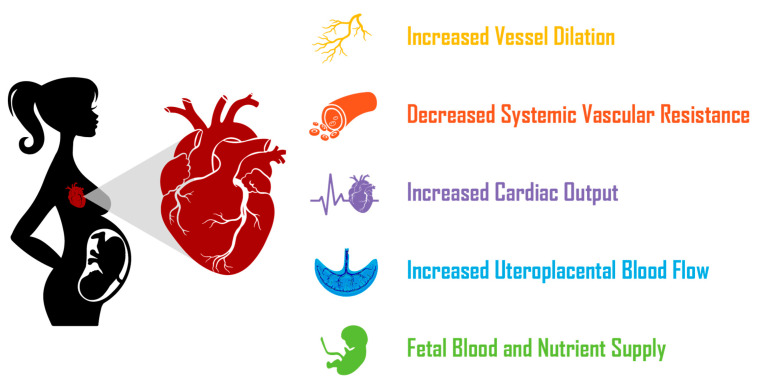
**Cardiovascular adaptation during pregnancy.** In pregnancy, cardiovascular adaptation leads to an increase in blood vessel dilation, an accompanying 20% decrease in systemic vascular resistance to accommodate the 40% increase in cardiac output. This adaptation thus increases uteroplacental blood flow and increases nutrient and blood supply to the fetus.

Numerous changes in the maternal cardiovascular system arise during a normal pregnancy including; increased cardiac output, augmented extracellular fluid volume, rise in arterial compliance, decrease in BP and a drop in total peripheral resistance [110] (Figure 3). Total peripheral resistance decreases through the first, second and third trimesters, whilst slowly increasing towards term [101]. Arterial compliance increases dramatically during the first trimester, remaining elevated throughout pregnancy [111]. Mean arterial blood pressure gradually falls as pregnancy progresses, with the largest drop occurring at 16 to 20 weeks (second trimester) and subsequently rising in the mid-third trimester [102]. During pregnancy, the change in BP is accompanied by an increase in blood flow to various organs to satisfy their increased metabolic needs, which leads to a dramatic increase in venous return and cardiac output [112]. Cardiac output gradually increases during the first trimester, with further increases occurring in the second trimester and then plateauing after 20 weeks gestation [102]. A significant increase in stroke volume and heart rate (HR) is closely associated with the increase in cardiac output [102]. 

An important factor that contributes significantly to cardiovascular adaptation during pregnancy, is nitric oxide (NO), which is synthesised in high amounts in normal pregnancy to mediate vasodilation [113]. The importance of adequate NO production cannot be overstated. Indeed, the inhibition of NO synthesis in pregnant rats, leads to an decrease in cardiac output and an increase in total peripheral resistance, which negatively impacts fetal blood and nutrient supply [102,113,114]. 

#### 3.2.1. Compromised Cardiovascular Adaptation in Pregnancy and the Associated Vascular Pathologies

Several complications during pregnancy involve alterations in the cardiovascular adaptation. For example, in PE, maternal and placental vasculature undergo alterations that pose a risk to maternal and fetal health. 

##### The Multifaceted Pathology of Preeclampsia (PE)

PE is one of the leading causes of mortality and morbidity affecting millions of pregnant women worldwide. PE is a complex and multifaceted disease, typically diagnosed in the 20th week of human pregnancy [115]. The diagnosis is confirmed by the onset of hypertension and vascular dysfunction in the presence of proteinuria, which can lead to multi-organ failure and death [116]. Moreover, PE pathology is associated with elevated NK cells and lymphocyte markers, which is indicative of an active innate and adaptive immune responses [117]. Although the cause of PE is unknown, the extensive chronic immune activation that accompanies PE results in endothelial inflammation, oxidative stress and dysfunction [118]. Endothelial dysfunction refers to an impairment of endothelial-dependent vasorelaxation, due to primarily a loss in NO bioavailability within the vessel wall [119]. NO production is determined by certain factors including endothelial nitric oxide synthase (eNOS) expression, intracellular calcium ([Ca^2+^]i) concentration, eNOS phosphorylation state [120] and vascular oxidative stress. The vascular dysfunction and hypertension associated with PE during pregnancy, negatively impact fetal development due to an impairment in nutrient and blood supply [116,121]. 

##### Gestational Hypertension/PIH as a Risk Factor for Preeclampsia

Gestational hypertension or pregnancy-induced hypertension (PIH) is characterised by increased BP of at least 140/90 mmHg, without significant, concurrent proteinuria, and is a risk factor for PE [122]. Although much of the aetiology of PIH is unknown, PIH can develop after 20 weeks of gestation, with the alterations in BP expected to normalise postpartum [123]. As gestation progresses, the incidence of PIH increases, with greater susceptibility demonstrated near term. Furthermore, PIH is thought to be an exaggerated form of maternal adaptation to the inherent cardiovascular changes. To date, PIH is not associated with adverse health outcomes for either mother or fetus, however PE is reported in about 15–20% of PIH associated pregnancies [123]. Severe PIH, which is defined as hypertension with a BP > 160/110 mmHg, is associated with an increased risk of adverse peri-natal outcomes including SGA and premature birth [124]. Investigation into the regulation of protein levels in the blood during the development of PIH may shed light on its role as a risk factor for PE.

## 4. The Link between Immune Dysregulation and Vascular Dysfunction

The inflammatory and oxidative damage that precede vascular dysfunction have been extensively studied. ROS production that overwhelms endogenous antioxidant systems is referred to as oxidative stress. Oxidative stress alters the redox state of the cytosol and/or biological compartments to become more oxidising [125]. The main culprits; O_2_^−^, OH-, ONOO- and H_2_O_2_, are known to contribute significantly to vascular dysfunction [126]. The build-up of oxidative stress promotes vascular disease via the ability of O_2_^−^ to react with vaso-protective NO; impairing NO signalling and promoting ONOO- formation. Moreover, pro-inflammatory cytokines such as TNF-α, and IL-6, and chemokines including macrophage inflammatory protein 1-alpha (MIP-1a) and monocyte chemoattractant protein-1(MCP-1), are known to significantly contribute to vascular dysfunction [127]. Macrophage/monocyte recruitment during the initial stages of vascular disease, results in the initiation of an inflammatory response via the activation/production of pro-inflammatory cytokines and ROS [128]. These macrophages also serve as antigen presenting cells that actively participate in the adaptive immune response and remodelling of the vasculature [128]. 

### 4.1. T Cells Are Critical in Vascular Inflammation and Dysfunction

The contributions of the innate and adaptive immune systems in CVD such as atherosclerosis and hypertension have been established [116,129,130]. While the innate immune response contributes significantly to arterial defence against an inflammatory challenge, it also plays a role in the initiation of CVD [131]. Furthermore, in CVD such as atherosclerosis, the T cells are suggested to contribute to the progression of atherosclerotic plaques, by elevating the inflammatory cells present in the vessel wall [129]. A comparable process involving T cells is demonstrated in hypertension [130]. Guzik et al., set out to decipher the role of T cells in the genesis of hypertension and vascular dysfunction. They found that in mice lacking B and T cells (RAG-1^−/−^ knockout mice), angiotensin II induced hypertension and vascular dysfunction was blunted. Moreover, these abnormalities were restored following adoptive transfer of T but not B cells [130]. The T cell populations were observed to be markedly increased in the periadventitial space of the aorta. In addition, T cell infiltration of the vasculature was associated with increases in the levels of adhesion proteins including intracellular adhesion molecule 1(ICAM)-(important in inflammation and immune response) and pro-inflammatory cytokine TNF-α. Importantly, this phenotype was accompanied by increased leukocyte activation in the perivascular adipose tissue (PVAT) [130]. Interestingly, in the absence of T cells, leukocytes failed to accumulate in the PVAT, which therefore suggests that the infiltration of T cells into the PVAT and adventitia of the vasculature, contribute to the overall inflammatory response that promotes vascular dysfunction in hypertension [132].

### 4.2. The Role of the Perivascular Adipose Tissue (PVAT) in the Vascular Immune Response and Inflammation

The PVAT in a normal state regulates vascular tone via the secretion of biologically active factors such as NO and adipose tissue-derived relaxing factor (ADRF) [133]. In a disease state, the PVAT becomes inflamed and the immune response that ensues contributes to oxidative stress, dampened NO bioavailability and vascular dysfunction [134]. Perivascular inflammation is common in vascular pathologies and its role in the immune and inflammatory response is evolving, with more involvement in vascular disease progression becoming evident [134]. In chronic diseases such as atherosclerosis, recent evidence has shed light on the role perivascular inflammation plays in various stages of the disease progression. Indeed, studies in atherosclerotic and low-density lipoprotein receptor knockout mouse models have demonstrated that a substantial amount of pro-inflammatory cytokines and chemokines are activated in the PVAT [135,136]. Also, the PVAT inflammation is believed to precede plaque formation, oxidative stress damage, and vascular dysfunction [137]. 

Immune cells including T cells, macrophages, dendritic cells, and adventitial tertiary lymphoid tissue, contribute to perivascular inflammation [138]. Both CD4^+^ and CD8^+^ T cells are present in high proportions in the PVAT, and are suggested to possess effector and memory T cell functions, that include Th1 (IFN-γ) and Th17 (TNF-a or IL-17) cells [134]. In a hypertensive mouse model, the percentage of circulating effector T cells expressing early immune activation marker; CD69, adhesion molecule; CD44, and inflammatory cytokines are increased and contribute to inflammation and vascular dysfunction [130,132]. Regulatory T cells (T_regs_) are a subset of T cells that are also present in the PVAT, where they express high levels of activation marker CD25 and fork-head transcription factor (FOXP3) to drive an anti-inflammatory phenotype, which helps maintain immune homeostasis and limit excessive immune responses within the PVAT [134]. 

Macrophages also accumulate predominantly in the PVAT and the adventitia during hypertension [139]. They are also involved in the release of superoxide radicals through the action of NOX2 oxidase [140]. The presence of infiltrating macrophages that produce pro-inflammatory cytokines such as TNF-α, IL-6 and IFN-γ contribute to PVAT inflammation. Furthermore, macrophages in the PVAT regulate T cell activation via antigen presentation, release of chemotaxis mediators and the expression of co-stimulatory ligands [128]. 

Dendritic cells function in regulating the adaptive immune response in CVD. DCs are capable of releasing mediators including IL-6 and IL-1β that can polarise T cells to produce pro-inflammatory cytokines such as TNF-α and IFN-γ, which are known to play significant roles in the genesis of hypertension and PVAT inflammation [130]. A positive feedback loop is suggested to occur in hypertension. Chronic oxidative stress resulting from T cell activation, enhances the adaptive immune function of DCs, leading to the formation of isoketal-protein adducts that can then accumulate in DCs to promote T cell activation [141,142]. 

### 4.3. Memory T Cell Formation, Accumulation, and Contribution to Vascular Dysfunction

Naïve and effector T cells differ from memory CD4^+^ and CD8^+^ T cells in multiple ways including their activation threshold and distinct effector functions of memory cells. Furthermore, distinct activation markers and intracellular proteins are expressed on memory cells, compared to naïve and effector T cells [143]. Memory T cells express unique adhesion receptors and chemokines that facilitate trafficking to infected tissues and organs [144]. In both humans and mice, naïve T cells express CD62L (L-selectin) and chemokine receptors including CCR7 facilitate recirculation of naïve T cells and routine immunosurveillance [145]. The migration of antigen presenting DCs from peripheral sites of infection to draining lymph nodes activates naïve T cells and prompts their differentiation into primary effector T cells, which acquire functional and migratory properties [144,146]. The differentiation of naïve T cells into effector T cells progresses through phases. These phases include I) the activation phase-where effector T cells can expand by over 1000-fold usually in cases of viral infection [147,148]. Studies employing major histocompatibility complex (MHC) class I tetramers in mice and humans have demonstrated that during the activation phase, about 50% of CD8^+^ T cells and about 10% of CD4^+^ T cells respond to a viral infection leading to mass expansion [149]. Although CD4^+^ and CD8^+^ T cells respond to viral infection, CD4^+^ T cells are unable to differentiate or expand as rapidly as CD8^+^ T cells [150]. The memory phase or final phase include the development of long-lasting memory T cells with antigen specificity [151]. Studies in mice suggest that the CD8^+^ memory T cells are derived from pools of dividing effector CD8^+^ T cells, and they express effector molecules such as IFN-γ [152,153]. 

In humans and mice, memory T cells develop into specific cell types including central memory T cells (T_CM_) and effector memory T cells (T_EM_) that all emerge from naïve T cells. The immune response to an infectious agent prompts increased antigen specificity and memory cell formation [154]. T_CM_ cell subsets express CD44^+^CD62L^+^ and differentiate into effector cells upon a secondary antigen encounter. The expression of CCR7 by T_CM_ cells favours their homing to secondary lymphoid organs (SLOs) such as the mediastinal lymph node (MLN) [155]. T_EM_ cells (CD44^+^CD62L^−^) home to peripheral tissues and are responsible for pro-inflammatory effector functions upon secondary antigen exposure [156]. In pre-eclampsia, T_CM_ cell levels are demonstrated to be comparable to levels in normal pregnancy. Moreover, in preeclamptic women, the proportion of CD4^+^ T_CM_ cells in peripheral blood were higher when compared to CD8^+^ T_CM_ cell populations [156,157]. A similar study comparing the proportion of T_EM_ cells in the peripheral blood of pregnant women with PE, observed a comparable level of CD4^+^ and CD8^+^ T_EM_ cell populations in both PE and their healthy controls [156,157]. In CVD such as atherosclerosis, CD4^+^ and CD8^+^ memory T cells contribute to endothelial vascular dysfunction and the progression of atherosclerosis [158]. In atherosclerotic ApoE^−/−^ mice, the vessel wall of the aorta was demonstrated to support T cell priming and naïve T cell trafficking. In addition, naïve T cells were reported to be primed by plaque antigens, leading to memory T cell development and atherosclerosis progression [158]. Similarly, Padgett et al. demonstrated that CD8^+^ naïve T cells, which differentiate into stem cell memory T cells, were significantly increased in humans and mice with atherosclerosis. Hence, stem cell memory T cells may contribute to the promotion of atherosclerosis progression, and therefore vascular dysfunction [159].

## 5. Extra-Pulmonary Dissemination of IAV and Vascular Inflammation

The dissemination mechanism of IAV into extra-pulmonary tissues is well established and has been validated in various mouse models [160,161]. For example, Haidari M et al. demonstrated that IAV viral RNA and its antigens disseminate into the aorta in wild type C57BL/6 and ApoE^−/−^ mice, following intranasal inoculation with a mouse adapted influenza A/HK (H3/N2) virus. Moreover, this led to a significant increase in chemokines and cytokines within the atherosclerotic plaque and the systemic circulation [160]. This study was the first to establish that IAV disseminates from the lung and infects the arterial wall to trigger an increase in cellular inflammation, which may disrupt atherosclerotic plaques and lead to a major cardiovascular event [160]. It is thought that direct infection of the arterial wall in non-atherosclerotic mice may also initiate an inflammatory reaction and lead to future atherosclerosis development, though further research is required. 

The association between IAV infection, dissemination to the arterial wall and disease progression has been established in individuals with CVD [160,161,162]. However, in pregnancy the association is yet to be clearly defined. It is known that pregnancy is a risk factor for developing severe pathology following IAV infection, nonetheless, little is known about whether IAV disseminates and infects the cardiovascular system in pregnancy, and how this may impact the fetus. Emerging evidence indicates, that in line with the local immune response, IAV also drives a systemic immune response [11,41]. Analogous to the cytokine storm, dissemination of IAV into systemic circulation results in a vascular inflammatory phenotype termed the “Vascular Storm” (Liong et al., 2020). The vascular storm consists of substantial molecular and cellular alterations in the maternal cardiovascular system that negatively impacts fetal development.

### The Vascular Storm Defined—IAV Infection Leads to Vascular Dysfunction in Pregnant Mice

There is limited information to what extent IAV influences the maternal cardiovascular system and this is despite the long list of maternal and fetal complications that arise to such infections. Therefore, the identification of an unappreciated respiratory, placental, and cardiovascular axis of pathology driven by IAV in pregnant mice by our group was paramount in defining the vascular storm. We demonstrated that the impact of IAV infection in pregnancy was beyond local lung pathology with a profound vascular disturbance that led to maternal endothelial and vascular dysfunction [77,78]. We proposed that the IAV-induced innate and adaptive immune response in the vasculature contributed to adverse fetal complications such as FGR. 

The “vascular storm” is a profound inflammatory response in the vasculature that is characterised by a substantial infiltration of innate and adaptive immune cells as well as pro-inflammatory cytokines [78]. Moreover, the innate immune response consisted of recruitment of endothelial-patrolling Ly6C^low^ monocytes, which are involved in focal necrosis of the endothelium in the event of infection [163]. This process occurs via TLR7 activation, which concomitantly recruits pro-inflammatory Ly6G neutrophils that function to increase viral clearance and initiate early T cell activation [78,163]. In addition, pro-inflammatory Ly6C^high^ monocytes are suggested to promote inflammatory responses in the aorta by differentiating into M2 macrophages that are likely to promote extracellular matrix remodelling [139]. The subsequent adaptive T cell response is characterised by an increase in activated CD4^+^ and CD8^+^ T cells in the aorta, and preferentially within the PVAT (Oseghale et al., 2022). Although IAV dissemination occurred in both pregnant and non-pregnant mice, the “vascular storm” phenotype was far more pronounced in pregnancy. A potential reason could be the level of IAV mRNA in the aorta in pregnant mice being significantly greater compared to the non-pregnant cohort [78]. A dysfunctional aorta in pregnancy is detrimental to maternal and fetal health, due to the fetus relying heavily on the optimal maternal major blood vessel function. The IAV-induced “vascular storm”, consisted of vascular dysfunction, altered pathways such as angiogenic markers, soluble FMS-like tyrosine kinase-1 (sFLT1) imbalance and the increase in highly inflammatory cell free fetal DNA (cffDNA) in maternal serum. Strikingly this resembled hallmarks of preeclampsia and therefore IAV infection might be a trigger for preeclampsia [78]. Furthermore, despite a lack in vertical transmission of IAV, the downstream influence of IAV-induced vascular dysfunction is demonstrated with placental and fetal growth retardation, which were associated with angiogenesis and hypoxia in the placenta and fetal brain. 

## 6. Conclusions

**Figure 4:**
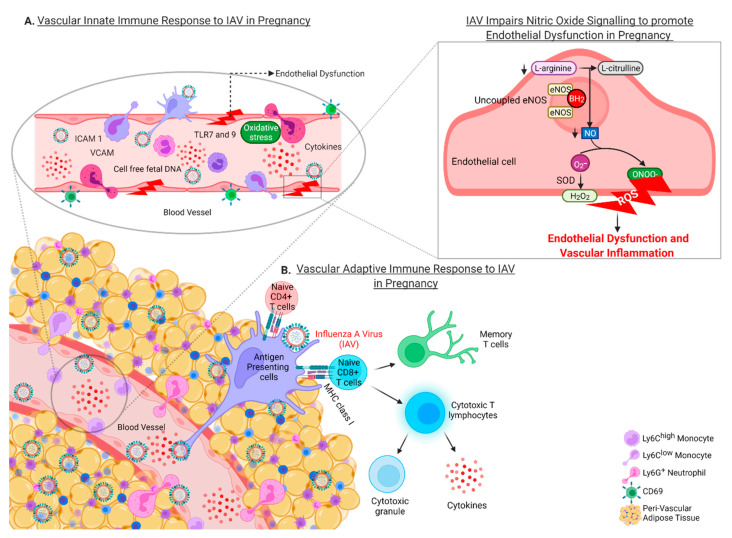
**Schematic summary diagram depicting the vascular innate and adaptive immune response to Influenza A virus (IAV) infection in pregnancy.** Following IAV dissemination from the lungs preferentially to the perivascular adipose tissue (PVAT) and then the vessel wall during pregnancy, the virus is sensed by single stranded RNA (ssRNA) toll like receptor 7 (TLR7) leading to the initiation of the “vascular storm” innate and adaptive immune response. The innate immune response is characterised by an increase in the recruitment of patrolling Ly6C^low^, pro-inflammatory Ly6C^high^ and Ly6G^+^ neutrophils to the vessel wall and the PVAT and in the process induces endothelial dysfunction and vascular inflammation. Pro-inflammatory cytokines and IAV triggers the maturation of antigen presenting cells (APCs) mainly in the PVAT to promote the adaptive immune response, with elevations in CD4^+^ and CD8^+^ T cells assisting to facilitate viral clearance, and consequently exacerbating vascular endothelial dysfunction and vascular inflammation. Endothelial dysfunction during IAV infection in pregnancy occurs following the uncoupling of endothelial nitric oxide synthase (eNOS) by IAV, which then leads to a reduction in L-arginine and consequently L-citrulline and nitric oxide (NO) bioavailability. Reduced NO triggers the generation of reactive oxygen species (ROS) including peroxynitrite (OONO^−^), superoxide (O_2_-) and hydrogen peroxide (H_2_O_2_), which contributes to vascular inflammation and endothelial dysfunction. In the process, following viral clearance, reservoirs of immunological memory and IAV-specific memory CD8^+^ T cells form in the aorta. The presence of these reservoirs is thought to initiate a positive feedback loop that further destabilises vascular function and may contribute to long-term cardiovascular disease.

The “vascular storm” (Figure 4) suggests that pregnant women run the risk of developing severe short and long-term cardiovascular consequences with dire outcomes for the mother and offspring. As this review has defined the cardiovascular implications of IAV infection in pregnancy, therapeutically, we need to consider treatment options that incorporate commercially available anti-viral drugs as well as drugs traditionally used for CVD. Although antiviral treatment options for pregnant women are classified as category C due to the limited clinical evidence to ensure safety in pregnancy, the CDC recommends antivirals such as oseltamivir phosphate and zanamivir within the first 48 h to limit viral replication and exacerbated systemic manifestations. However, we need to consider treatments for CVD that are safe during pregnancy such as vasodilators (hydralazine) and antiplatelet drugs (low dose Aspirin), both of which are used in PE. Given the similarities between the vascular storm triggered by IAV and pre-eclampsia, the combination of antiviral medications and CVD medications may assist in limiting viral replication and preventing virus dissemination to the cardiovascular system and its dysfunction. Finally, the cardiovascular manifestations of IAV infection suggest that a change in the clinical management of IAV infection in pregnancy is needed including the implementation of routine follow up programs to assess the vascular health of women. 

## Data Availability

Not applicable.
